# Anaemia and kidney dysfunction in Caribbean Type 2 diabetic patients

**DOI:** 10.1186/1475-2840-7-25

**Published:** 2008-08-27

**Authors:** Chidum E Ezenwaka, Altheia Jones-LeCointe, Emeka Nwagbara, Dawn Seales, Fidelis Okali

**Affiliations:** 1 Unit of Pathology & Microbiology, Faculty of Medical Sciences, The University of the West Indies, St Augustine, Trinidad and Tobago; 2 Tobago Regional Health Authority, Trinidad and Tobago

## Abstract

**Background:**

Anaemia has been shown in previous studies to be a risk factor for cardiovascular disease in diabetic patients with chronic kidney disorder. This study was aimed to assess the prevalence of anaemia and kidney dysfunction in Caribbean type 2 diabetic patients that have been previously shown to have a high prevalence of the metabolic syndrome.

**Methods:**

155 type 2 diabetic patients and 51 non-diabetic subjects of African origin were studied. Anthropometric parameters were measured and fasting blood samples were collected for glucose, creatinine, glycated hemoglobin and complete blood count. Anaemia was defined as haemoglobin < 12 g/dl (F) or < 13 g/dl (M). Kidney function was assessed using glomerular filtration rate (GFR) as estimated by the four-variable Modification of Diet in Renal Disease (MDRD) study equation. Subjects were considered to have chronic kidney disease when the estimated GFR was < 60 ml/min per 1.73 m^2^. Comparisons for within- and between-gender, between diabetic and non-diabetic subjects were performed using Student's t-test while chi-square test was employed for categorical variables.

**Results:**

The diabetic patients were older than the non-diabetic subjects. While male non-diabetic subjects had significantly higher red blood cell count (RBC), haemoglobin and hematocrit concentrations than non-diabetic female subjects (p < 0.001), the RBC and hematocrit concentrations were similar in male and female diabetic patients. Furthermore, irrespective of gender, diabetic patients had significantly higher prevalence rate of anemia than non-diabetic subjects (p < 0.05). Anaemic diabetes patients had significantly lower GFR (67.1 ± 3.0 vs. 87.9 ± 5.4 ml/min per 1.73 m^2^, p < 0.001) than non-anaemic patients.

**Conclusion:**

A high prevalence of anaemia was identified in this group of type 2 diabetic patients previously shown to have a high prevalence of the metabolic syndrome. It is therefore recommended that diagnostic laboratories in developing countries and elsewhere should include complete blood count in routine laboratory investigations in the management of diabetic patients.

## Introduction

Trinidad and Tobago has high prevalence of diabetes mellitus that the International Diabetes Federation has projected that by the year 2025, 11.8% of the population will be diagnosed with type 2 diabetes [1]. Although the projected prevalence rate represents one of the highest prevalence rates in the North American region [1], of greater concern are the several reports of poor glycaemic control amongst type 2 diabetic patients at the primary care settings in this population [2-4]. Poor glycaemia control, obesity, sedentary lifestyle etc are some of the factors that have been implicated in the increased risk of cardiovascular disease amongst diabetic patients in this population [2,5]. Unfortunately, most of the published data from this population have not assessed the impact of anaemia in the risk of cardiovascular disease in type 2 diabetic patients [2-5]. Yet, there are pathophysiologic reasons why the presence of anaemia may lead to adverse cardiovascular consequences especially in diabetic patients. For instance, it has been demonstrated that patients with chronic anaemia had a high cardiac output and a low systemic vascular resistance [6]. In the long term, this may result in maladaptive left ventricular hypertrophy (LVH), which is a known risk factor for adverse cardiovascular outcome and all-cause mortality [7,8]. Furthermore, anaemia has been shown to be a risk factor for adverse cardiovascular outcomes in non-diabetic [9,10] and diabetic patients [11] with chronic kidney disease. Therefore, given that diabetes is a leading cause of kidney disease and kidney failure, assessment of anaemia and kidney dysfunction in diabetic patients should be a regular laboratory tests. This is particularly important given that anaemia presents earlier and often more severe in diabetic patients with chronic kidney disease compared with chronic kidney disease patients without diabetes [12,13]. Although there is scanty reports on the prevalence of kidney disease in type 2 diabetic patients in this population, report of end-stage renal disease that presented with chronic anaemia has been documented [14]. Thus, the presence of anemia in diabetic patients with undetected renal dysfunction may be particularly dangerous especially at the primary care setting where routine laboratory investigations are infrequent and haematological tests not usually included as part of the laboratory tests for the patients' management. In this regard, the present study is aimed to assess the prevalence of anaemia and kidney dysfunction in a cross-section of Afro-Caribbean type 2 diabetic patients that were previously shown to have a high prevalence of the metabolic syndrome [15].

## Subjects and methods

### Type 2 diabetic patients

One hundred and fifty-five (46 males, 109 females) type 2 diabetic patients visiting, consecutively, eight lifestyle disease clinics at primary care setting in Tobago (between June and November 2006) participated in the study. Patients were considered as type 2 diabetic if they had been managed on oral hypoglycaemic medication and/or diet/exercise since diagnosis (except on occasions when patients took insulin to control hyperglycaemia). The protocol was as previously published [15]. Briefly, the patients received information leaflets and posters explaining the objectives and protocol of the research study and a member of the research group addressed the patients during the clinics to explain the rationale for the study. Subsequently, the Research Assistant or Clinic Nurse took names and other information of patients who volunteered to participate in the study. The patients were subsequently reminded of the importance of overnight fasting a day preceding the study.

### Non-diabetic subjects

Fifty-one (22 males, 29 females) apparently healthy non-diabetic subjects living in the same environment/city (Tobago) with the diabetic patients qualified to participate in the study as non-diabetic control subjects. Each volunteer underwent compulsory oral glucose tolerance tests (OGTT, 75 g anhydrous glucose in 250–300 ml water) to detect undiagnosed diabetes (fasting plasma glucose > 7.0 with 2-hour plasma glucose > 11.1 mmol/L) and impaired glucose tolerance (2-hour plasma glucose > 7.8 mmol/L) [16]. Subjects with plasma glucose values diagnostic of diabetes were excluded from the study and data analysis while subjects with plasma glucose values diagnostic of IGT were included in the study and data analysis.

### Study protocol

Informed consent was obtained from both the diabetic and non-diabetic subjects that participated in the study. The study protocol conforms to the ethical guidelines of the 1975 Declaration of Helsinki as reflected in a *priori *approval by our institutional Ethics Review Committee. All subjects were studied at the clinics in the morning after an overnight (12–14 hours) fast. Details of self-reported ethnic origin and age were directly ascertained from the patients and recorded. Then, waist (cm), at the level of the umbilicus with the patient standing and breathing normally, and hip circumferences (cm), at the level of the largest projection of the buttocks, were obtained by tape measure while weight (kg), with standard hospital balance, and height (m), with metal rule, were measured (in light clothing, without shoes). Then, fasting blood sample was collected from each subject and, for the non-diabetic subjects alone, 75 g anhydrous glucose in 250–300 ml water was given to each subject and blood samples collected every 30 minutes for 120 minutes. Blood samples were preserved in appropriate tubes for complete blood count, creatinine, glucose and glycated haemoglobin measurements.

### Laboratory analysis

Plasma glucose and serum creatinine were measured in multi-channel auto-analysers using dry slide kits (Johnson & Johnson Vitros 250, Ortho-Clinical Diagnostics Inc., Rochester NY, USA). Glycated haemoglobin (HbA_1c_) was determined using boronate affinity assay (Axis-Shield PoC AS, N-0504 Oslo, Norway) while complete blood count was measured using the routine Coulter counter machine (Sysmex XE 2100, Germany).

#### Definitions, calculations and statistics

The results are expressed as mean ± SE. The Statistical Package for the Social Sciences (SPSS Inc., Chicago, USA) software was used in all analyses. Anemia was defined as haemoglobin < 12 g/dl in females and < 13 g/dl in males [17]. Kidney function was assessed using glomerular filtration rate (GFR) as estimated by the four-variable Modification of Diet in Renal Disease (MDRD) study equation as follows: GFR = 186.3 × (serum creatinine^-1.154^) × (age^-0.203^) × 1.212 (if black) × 0.742 (if female). GFR was expressed in ml/min/1.73 m^2 ^[18] and patients were considered to have chronic kidney disease when the estimated GFR was < 60 ml/min per 1.73 m^2 ^[11]. Comparisons within- and between-gender, between diabetic and non-diabetic subjects were performed using Student's t-test while chi-square test was employed for categorical variables. A p-value < 0.05 was considered statistically significant on two-tailed testing for all analysis.

## Results

Table 1 shows the clinical characteristics of all the subjects studied. The diabetic patients were significantly older than the non-diabetic subjects (p < 0.001). Compared with non-diabetic subjects, lower percentage of the diabetic patients smoke cigarettes and drink alcoholic beverages (Table 1, p < 0.05). The male non-diabetic subjects had significantly higher red blood cell count (RBC), hemoglobin (Hb) and hematocrit levels than their diabetic counterparts (Table 2, p < 0.001). However, the female diabetic and non-diabetic subjects had similar Hb concentrations (12.1 ± 0.1 vs. 12.5 ± 0.2 g/dl, p > 0.05). While male non-diabetic subjects had significantly higher RBC, Hb and hematocrit than non-diabetic female subjects (p < 0.001), the RBC and hematocrit concentrations were similar in male and female diabetic patients (Table 2, p > 0.05). The prevalence of anemia and chronic kidney disease in the subjects are shown on Table 3. Irrespective of gender, diabetic patients had significantly higher prevalence rate of anemia than non-diabetic subjects (Table 4, p < 0.05). Again, diabetic patients had significantly higher prevalence of kidney dysfunction compared with the non-diabetic subjects (Table 4, p < 0.05). The diabetic patients with anemia had significantly higher serum creatinine levels (1.4 ± 0.1 vs. 1.0 ± 0.03 mg/dl, p < 0.001) and lower GFR (67.1 ± 3.0 vs. 87.9 ± 5.4 ml/min per 1.73 m^2^, p < 0.001) than diabetic patients without anemia. Similarly, diabetic patients with anaemia had significantly higher levels of glycated hemoglobin (index of blood glucose control), creatinine and higher prevalence of kidney dysfunction than non-diabetic subjects with anaemia (Table 3, p < 0.05).

**Table 1:** Clinical characteristics of the diabetic and non-diabetic subjects studied

**Variables**	**Type 2 diabetic patients**	**Non-diabetic subjects**
Gender (m/f ratio)	46/109	22/29
Age (yr)	65.9 ± 0.9	48.8 ± 1.2**
Duration of diabetes (yr)	10.6 ± 0.7	-
Weight (kg)	77.9 ± 1.2	83.3 ± 2.0*
Height (m)	1.64 ± 0.01	1.68 ± 0.01*
Body mass index (kg/m^2^)	28.6 ± 0.4	30.2 ± 0.8
Waist circumference (cm)	99.1 ± 1.0	95.5 ± 1.4
Hip circumference (cm)	105.0 ± 0.8	108.8 ± 1.4*
Unemployed (%)	112 (72.3)	3 (5.9)**
Cigarette smokers (%)	6 (3.9)	6 (11.8)*
Alcohol users (%)	29 (18.7)	23 (45.1)**

*P < 0.05, **P < 0.001 for comparisons between diabetic and non-diabetic subjects

**Table 2:** Between-gender comparison of the haematological tests of the diabetic and non-diabetic subjects

Haematology profile	**Type 2 diabetic patients**		**Non-diabetic subjects**	
	**Males**	**Females**	**Males**	**Females**
Age (years)	^§^68.9 ± 1.2	64.6 ± 1.2^¶^	48.9 ± 1.4	48.8 ± 1.8^╫╫^
Whole Blood Count (× 10^3^/uL)	5.5 ± 0.3	6.0 ± 0.1	5.5 ± 0.2	5.5 ± 0.3
Red Blood Cell (× 10^6^/uL)	^§§^4.6 ± 0.1	4.4 ± 0.1	5.1 ± 0.1**	4.6 ± 0.1^╫^
Haemoglobin (g/dl)	^§§^12.9 ± 0.2	12.1 ± 0.1^¶^	14.6 ± 0.3**	12.5 ± 0.2
Hematocrit (%)	^§^39.4 ± 0.5	37.5 ± 0.3	44.2 ± 0.7**	39.0 ± 0.5^╫^
Mean Corpuscular Volume (FL)	87.0 ± 0.7	85.3 ± 0.6^¶^	87.4 ± 1.4	84.4 ± 1.3
Mean Corpuscular Haemoglobin (pg)	28.5 ± 0.3	28.2 ± 0.6	28.8 ± 0.5*	27.1 ± 0.5*
Platelet Count (× 10^3^/uL)	224.2 ± 9.9	244.4 ± 5.7	237.9 ± 13.8	262.2 ± 10.1
Mean Platelet Volume (Fl)	11.1 ± 0.1	11.1 ± 0.1	11.2 ± 0.2	10.9 ± 0.2

*P < 0.05, **P < 0.001 for comparisons between male and female non-diabetic subjects

^¶^P < 0.05 for comparisons between male and female diabetic patients

^§^P < 0.05, ^§§^P < 0.001 for comparisons between male diabetic and male non-diabetic subjects

^╫^P < 0.05, ^╫╫^P < 0.001 for comparisons between female diabetic and female non-diabetic subjects

**Table 3:** Glycaemic control and levels of kidney function in patients with and without anemia

	**Type 2 diabetic patients**		**Non-diabetic subjects**	
	*Anemia* *N = 72*	*No Anemia* *N = 83*	*Anemia* *N = 8*	*No Anemia* *N = 43*
Glycated hemoglobin (%)	7.7 ± 0.3	7.6 ± 0.2	5.7 ± 0.2^¶^	5.9 ± 0.2
Fasting plasma glucose (mmol/L)	9.3 ± 0.6	9.1 ± 0.4	4.6 ± 0.1^¶^	5.0 ± 0.2
Creatinine (mg/dl)	1.4 ± 0.1	1.0 ± 0.03**	1.0 ± 0.1^¶^	1.1 ± 0.02
Glomerular filtration rate (ml/min/1.73 m^2^) (index of kidney function)	67.1 ± 3.0	87.9 ± 5.4**	86.2 ± 5.1^¶^	82.6 ± 2.1
Chronic kidney disease (%) (estimated GFR < 60 ml/min per 1.73 m^2^)	27 (38.6)	9 (10.8)**	1 (12.5)	1 (2.4)

**P < 0.001 for comparisons between diabetic patients with and without anaemia

^¶^P < 0.05 for comparisons between diabetic patients with anaemia and non-diabetic subjects with anaemia

**Table 4:** Prevalence of Anemia and chronic kidney disease (estimated by glomerular filtration rate) in diabetic and non-diabetic subjects

	**Type 2 diabetic patients**		**Non-diabetic subjects**	
	*Males*	*Females*	*Males*	*Females*
Anemia (%) (Hb < 12 g/dL [F] or < 13 g/dl [M])	22 (47.8)	50 (45.9)	2 (9.1)*	6 (20.7)^¶^
Creatinine (mg/dl)	1.6 ± 0.2	1.0 ± 0.03	1.2 ± 0.04	0.96 ± 0.03
Glomerular filtration rate (ml/min/1.73 m^2^) (index of kidney function)	71.9 ± 2.7	81.0 ± 4.5	83.9 ± 3.1*	82.6 ± 2.5
Chronic kidney disease (%) (estimated GFR < 60 ml/min per 1.73 m^2^)	12 (26.7)	24 (22.2)	1 (4.5)**	1 (3.6)^¶^

*P < 0.05, **P < 0.001 for comparisons between male diabetic and non-diabetic subjects

^¶^P < 0.05 for comparisons between female diabetic and non-diabetic subjects.

## Discussion

This study has shown that anaemia was more prevalent in diabetic than non-diabetic subjects irrespective of gender, and diabetic patients with anaemia had the lowest kidney function compared with patients without anaemia or non-diabetic subjects with anaemia. The implications of these findings in relation to diabetes management are further discussed.

All the new frontiers in the management of type 2 diabetes are aimed at achieving optimal blood glucose control so as to prevent macro- and micro-vascular complications [19-22]. In the primary care setting in the clinics studied, assessment of haematological parameters is no part of routine diabetic evaluation. Therefore, the finding in this study of high prevalence rates of anaemia amongst the diabetic patients studied, irrespective of gender, is perhaps, the first report in the Caribbean diabetic population. This finding is significant given that anaemia has previously been shown to be associated with cardiovascular disease and all-cause mortality in diabetic patients [11]. However, other workers have reported an association between type 2 diabetes and elevated haematocrit, which is thought to negatively affect nitric oxide availability resulting in cardiovascular disease [23]. Furthermore, increase of whole blood viscosity due to increased levels of haematological parameters such as fibrinogen has been reported in type 2 diabetic patients with arteriosclerosis obliterans [24]. We are not aware of any study at the primary care settings in the Caribbean population that evaluated the prevalence of anaemia, high haematocrit or increased whole blood viscosity in individuals with type 2 diabetes. In contrast to the elevated haematocrit report [23], our study found high prevalence rates of anaemia in diabetic patients, irrespective of gender. This finding may be indicative of early renal disease given that previous studies showed earlier appearance of anaemia in diabetic patients; with an inverse relationship between creatinine concentration and haemoglobin levels [12,13]. However, it should be noted that macro-vascular, and not micro-vascular, complication is commoner in type 2 diabetes [19] and as such there appears to be little or no documented report of kidney complications in type 2 diabetes in our population. Although there was a report of two Afro-Caribbean teenagers with end-stage renal disease who presented in clinics with anaemia [14], incidence or prevalence of diabetic patients with anaemia are scanty.

In this study, anaemia was more prevalent amongst the diabetic patients than non-diabetic subjects and the former group of subjects were previously shown to have a high prevalence of the metabolic syndrome [15]. The limitation is that the study was not designed to determine the type of anaemia the patients had, though iron-deficiency anaemia is common in some other developing countries [25,26]. Thus, diabetic patients and the senior citizens, with limited food choices, would be more vulnerable to iron-deficiency anaemia and all cause mortality [11]. The findings in the present study have implications for diabetes management in that they appear to indicate a need for routine full blood counts in primary care diabetes management. Intervention with erythropoietin has been shown to improve the quality of life for anaemic patients in chronic renal failure [25]. For diabetic patients, it is well documented that reducing blood glucose levels and targeting acceptable glycated haemoglobin (HbA1c) levels have been the focus so as to prevent the risk of micro- and macro-vascular complications [19,27,28]. Even then, the reality is that most patients with type 2 diabetes in developed and developing countries have glycated haemoglobin levels above the recommended target levels and are prone to macro-vascular complications [2,3]. Inadequate or absence of laboratory facilities at the primary care settings in many developing countries is a major limitation in routine laboratory assessment of diabetic patients. We believe that early detection and management of anaemia in diabetic patients at the primary care setting would be cost effective in so far as it would reduce hospital admissions and maintain optimum health. This could be achieved through the provision of adequate laboratory facilities and expansion of the scope of laboratory investigations used in the management of diabetic patients. Therefore, it is suggested that all diagnostic laboratories in developing countries and elsewhere should include complete blood count as one of the routine laboratory tests required in the management of diabetic patients.

## Competing interests

The authors declare that they have no competing interests.

## Authors' contributions

CEE – involved in conception, design, acquisition of data, data analysis, interpretation and drafting of manuscript. AJ–L – involved in conception, interpretation and drafting of manuscript. EN – acquisition of data, interpretation and drafting of manuscript. DS – acquisition of data, data analysis, interpretation and drafting of manuscript. FO – acquisition of data, interpretation and drafting of manuscript.

## References

[B1] International Diabetes Federation (2003). Diabetes Atlas.

[B2] Ezenwaka CE, Offiah NV (2001). Differences in glycemic control and cardiovascular risk in primary care patients with type 2 diabetes in West Indies. Clin Exp Med.

[B3] Ezenwaka CE (2003). Metabolic control of type 2 diabetic patients commonly treated with sulphonylureas in a developing country. East Afr Med J.

[B4] Ezenwaka CE, Kalloo R (2004). Postprandial glucose control in Type 2 diabetic patients visiting two different primary care clinics in Trinidad, West Indies. West Indian Med J.

[B5] Ezenwaka CE, Offiah NV (2002). Abdominal obesity in Type 2 diabetic patients visiting primary healthcare clinics in Trinidad, West Indies. Scand J Primary Health Care.

[B6] Anand IS, Chandrashekhar Y, Ferrari R, Poole-Wilson PA, Harris PC (1993). Pathogenesis of oedema in chronic severe anaemia: Studies of body water and sodium, renal function, haemodynamic variables and plasma hormones. Br Heart J.

[B7] Al-Ahmad A, Rand WM, Manjunath G, Konstam MA, Salem DN, Levey AS, Sarnak MJ (2001). Reduced kidney function and anemia as risk factors for mortality in patients with left ventricular dysfunction. J Am Coll Cardiol.

[B8] Elhendy A, Modesto KM, Mohoney DW, Khandheria BK, Seward JB, Pellikka PA (2003). Prediction of mortality in patients with left ventricular hypertrophy by clinical, exercise stress and echocardiographic data. J Am Coll Cardiol.

[B9] Foley RN, Parfrey PS, Harnett JD, Kent GM, Murray DC, Barre PE (1996). The impact of anemia on cardiomyopathy, morbidity and mortality in end-stage renal disease. Am J Kidney Dis.

[B10] Levin A (2002). Anemia and left ventricular hypertrophy in chronic kidney disease populations. A review of the current state of knowledge. Kidney Int.

[B11] Vlagopoulos PT, Tighiouart H, Weiner DE, Griffith J, Pettitt D, Salem DN, Levey AS, Sarnak MJ (2005). Anemia as a risk factor for cardiovascular disease and all-cause mortality in diabetes: The impact of chronic kidney disease. J Am Soc Nephrol.

[B12] Deray G, Heurtier A, Grimaldi A, Launay Vacher V, Isnard Bagnis C (2004). Anemia and diabetes. Am J Nephrol.

[B13] Thomas S, Rampersad M (2004). Anaemia in diabetes. Acta Diabetol.

[B14] Balkaran B, Ramcharan J, Rao AV, Ramanjaneyulu M, Roberts LA (1998). Two Afro-Trinidadian siblings with end-stage renal disease. Ann Trop Paediatr.

[B15] Ezenwaka CE, Nwagbara E, Seales D, Okali F, Hussaini S, Raja Bn, Sell H, Avci H, Eckel J (2007). A comparative study of the prevalence of components of the metabolic syndrome in type 2 diabetic patients in two Caribbean Islands using the New International Diabetes Federation definition. Arch Physiol Biochem.

[B16] The DECODE Study Group (1999). Glucose tolerance and mortality: comparison of WHO and American Diabetes Association Diagnostic criteria. Lancet.

[B17] World Health Organisation (WHO) (1968). Nutritional anaemias. Report of a WHO scientific group. World Health Organisation Tech Rep Ser 405.

[B18] Levey AS, Bosch JP, Lewis JB, Greene T, Rogers N, Roth D (1999). A more accurate method to estimate glomerular filtration rate from creatinine: A new prediction equation. Modification of Diet in Renal Disease Study Group. Ann Intern Med.

[B19] Stratton IM, Adler AL, Neil HA, Matthews DR, Manley SE, Cull CA (2000). Association of glycaemia with macrovascular and microvascular complications of type 2 diabetes (UKPDS 35): prospective observational study. BMJ.

[B20] Barnett AH (2004). Treating to goal: challenges of current management. Eur J Endocrinol.

[B21] Mancuso M, Ingegnosi C, Caruso-Nicoletti M (2005). Self monitoring blood glucose and quality of care. Acta Biomed.

[B22] Mudaliar S (2007). New frontiers in the management of type 2 diabetes. Indian J Med Res.

[B23] Natali A, Toschi E, Baldeweg S, Casolaro A, Baldi S, Sironi AM, Yudkin JS, Ferrannini E (2005). Haematocrit, type 2 diabetes, and endothelium-dependent vasodilatation of resistance vessels. Eur Heart J.

[B24] Tkác I, Tkácová R, Takác M, Lazúr J (1992). Hematologic changes in type 2 diabetic patients with various localizations of peripheral vascular disease. Vasa.

[B25] Akinsola A, Durosinmi MO, Akinola NO (2000). The haematological profile of Nigerians with chronic renal failure. Afr J Med Med Sci.

[B26] Nojilana B, Norman R, Dhansay MA, Labadarios D, van Stuijvenberg ME, Bradshaw D, South African Comparative Risk Assessment Collaborating Group (2007). Estimating the burden of disease attributable to iron deficiency anaemia in South Africa in 2000. S Afr Med J.

[B27] DCCT Research Group (1993). The effect of intensive treatment of diabetes on the development and progression of long-term complications in insulin-dependent diabetes mellitus. N Engl J Med.

[B28] UKPDS (1998). Intensive blood glucose control with sulphonylureas or insulin compared with conventional treatment and risk in of complications in patients with type 2 diabetes. Lancet.

